# A Case of Traumatic Ventral Hernia

**Published:** 1875-11

**Authors:** 


A Case of Traumatic Ventral Hernia.—E. S., fifty-eight years of age, was admitted into Bellevue Hospital May 25th, 1875, and gave the following history:

He was working in a factory, filling metallic plates, and found it very convenient when at work to rest the material to be worked on the bench, and hold it firmly by pressing against it with his abdomen. This practice (he being a new hand and working very hard) made him very tender and sore in the abdominal region. In the month of October, 1874—shortly after beginning this work—he found a circumscribed point of tenderness in the abdomen, midway between the umbilicus and pubes, and in the median line. The following day it became worse, and in a few days ended in an abscess, which was opened by a physician. With the pus which followed came a few inches of intestine. At the end of six weeks he had recovered and resumed work at his trade.

On the morning of the day of admission he was working at a machine run by hand. Projecting from the front of this machine, about three feet from the floor, was a dull piece of iron, in the form of a spike. Against this the patient, while suddenly leaning over struck his abdomen, causing a rupture of the walls, and a protrusion of intestines.

On admission, patient suffered considerably from shock and felt very weak. Before being admitted to this hospital several attempts had been made at reduction of the hernia, without success—once under the influence of an anæsthetic. He had also taken a hot bath. Examination of abdominal walls showed a loop of intestines, thirty-five or forty inches long, protruding from an opening about an inch and a half in length. This opening has taken place in the cicatricial tissue, in the site of an old abscess, which extends from the umbilicus downwards, in the median line, to the pubes. The wound is situated lying in a vertical direction, and occupying a position half way between these two points. The intestine is deeply congested; and at one point, near the extremity of the loop, there is a dark discoloration, which looks very much like gangrene. Bloody serum exudes from the surface of the intestine.

The patient was anæsthetized, and after the gut was washed, an attempt was made to reduce, which was ineffectual, owing to the swollen condition of the intestines, and the cicatricial rigidity at the opening. The upper part of the wound was made larger by the introduction of a hernia knife—care being taken to thrust the finger in between the peritoneum and abdominal wall, so as to avoid injury to the former—making an incision about an inch long; and after stretching the wound with the fingers, the hernia was reduced. The wound was then closed by silver wire sutures, and the patient kept moderately under the influence of opium for four or five days. He was then discharged cured in eighteen days, with orders to wear an abdominal support.—*Med. Record.*

